# System Design for Sensing in Manufacturing to Apply AI through Hierarchical Abstraction Levels

**DOI:** 10.3390/s24144508

**Published:** 2024-07-12

**Authors:** Georgios Sopidis, Michael Haslgrübler, Behrooz Azadi, Ouijdane Guiza, Martin Schobesberger, Bernhard Anzengruber-Tanase, Alois Ferscha

**Affiliations:** 1 Pro2Future GmbH, Altenberger Strasse 69, 4040 Linz, Austria; michael.haslgruebler@pro2future.at (M.H.); behrooz.azadi@pro2future.at (B.A.); ouijdane.guiza@pro2future.at (O.G.); bernhard.anzengruber@pro2future.at (B.A.-T.); 2 Institute of Pervasive Computing, Johannes Kepler University, Altenberger Straße 69, 4040 Linz, Austria; martin.schobesberger@jku.at (M.S.); ferscha@pervasive.jku.at (A.F.)

**Keywords:** activity recognition, manual industrial assembly, hierarchical taxonomy, human awareness, activity abstraction levels, artificial intelligence, system design

## Abstract

Activity recognition combined with artificial intelligence is a vital area of research, ranging across diverse domains, from sports and healthcare to smart homes. In the industrial domain, and the manual assembly lines, the emphasis shifts to human–machine interaction and thus to human activity recognition (HAR) within complex operational environments. Developing models and methods that can reliably and efficiently identify human activities, traditionally just categorized as either simple or complex activities, remains a key challenge in the field. Limitations of the existing methods and approaches include their inability to consider the contextual complexities associated with the performed activities. Our approach to address this challenge is to create different levels of activity abstractions, which allow for a more nuanced comprehension of activities and define their underlying patterns. Specifically, we propose a new hierarchical taxonomy for human activity abstraction levels based on the context of the performed activities that can be used in HAR. The proposed hierarchy consists of five levels, namely atomic, micro, meso, macro, and mega. We compare this taxonomy with other approaches that divide activities into simple and complex categories as well as other similar classification schemes and provide real-world examples in different applications to demonstrate its efficacy. Regarding advanced technologies like artificial intelligence, our study aims to guide and optimize industrial assembly procedures, particularly in uncontrolled non-laboratory environments, by shaping workflows to enable structured data analysis and highlighting correlations across various levels throughout the assembly progression. In addition, it establishes effective communication and shared understanding between researchers and industry professionals while also providing them with the essential resources to facilitate the development of systems, sensors, and algorithms for custom industrial use cases that adapt to the level of abstraction.

## 1. Introduction

Industrial manufacturing and assembly processes are fundamental to modern society, driving innovation, economic growth, and technological advancement [1]. These procedures rely heavily on human expertise and involve manual assembly tasks and activities [2,3]. Despite the prevailing focus on locomotion activities in human activity recognition (HAR), it is necessary to recognize that human industrial activities involve complexities far beyond locomotion activities, which need to be explored. In this field, the efficient execution of activities is essential for achieving production goals, ensuring product quality, and maximizing operational efficiency. However, understanding the numerous activities involved in industrial processes poses significant challenges, requiring sophisticated approaches for analysis, classification, and optimization.

At the core of industrial operations lie the activities performed by individuals or individuals in combination with machines or automated systems [4]. According to the authors of [5], manual assembly tasks have a clear distinction regarding complexity. Objective assembly complexity refers to inherent properties of the assembly process, such as the number and dependencies of components. In contrast, perceived assembly complexity is subjective and influenced by personal capabilities and experience. From the manipulation of individual components to the orchestration of complex production lines, each activity plays a crucial role in shaping the outcome of the manufacturing process. Yet, the complexity and diversity of these activities present difficulties for researchers, engineers, and workers in the field who want to understand them better to enhance performance.

Traditional approaches for activity analysis have often relied on recognizing few activities and in simplistic categorizations, such as simple versus complex activities [6,7]. Simple activities, such as walking, running, sitting, or standing, are extensively researched, whereas everything else is categorized as complex. Consequently, the recognition of complex activities remains relatively unexplored, as highlighted by the authors in [8]. While these frameworks provide a basic understanding of activity complexity, they prove ineffective in capturing the nuanced structure and hierarchical organization of industrial tasks or miss the depth and detail needed to analyze and categorize industrial activities. Activities can be categorized along a scale of increasing complexity, as discussed by Schneider et al. [9], where simple activities occur for a short period, while complex activities may occur for more prolonged periods. Furthermore, Peng et al. [10] state that the features designed for simple activities are poor at representing complex activities.

Our work proposes a novel hierarchical activity abstraction framework tailored to the specific level of abstraction, ensuring that the activity model fits the task in industrial contexts. Within this framework, distinguishing between activity abstractions becomes critical since, e.g., distinct statistical, temporal, and spatial properties appear at different levels of abstraction, which could lead to more efficient sensor placement, information capture, feature extraction, resource utilization, and architecture design.

Our approach contributes to industrial activity analysis and optimization by examining the structure of industrial production and assembly processes to enable an understanding of the wide range of involved activities and their role in complex operational environments. In that regard, we introduce an activity abstraction framework that acknowledges factors such as complexity, granularity, and hierarchical organization, providing a structured approach to activity analysis. Additionally, we recommend suitable AI-guided solutions for optimizing industrial processes, ranging from real-time activity monitoring, which forms the core of our analysis, to broader areas such as predictive maintenance and adaptive control strategies. Finally, our work intends to enable the development of recognition technologies and the awareness of industrial assembly in uncontrolled environments, allowing researchers and practitioners to design systems, sensors, and algorithms aligned with the required level of abstraction.

The following sections outline the structure of the paper: Section 2 provides a review of related work. Section 3 introduces an industrial assembly process and proposes our taxonomy framework. In Section 4, we compare our model with the state of the art by applying our and the existing approaches to a real industrial use case. Section 5 presents the application of our taxonomy to develop AI systems for custom industrial use cases, recommending necessary factors and variables to consider during the design process in addition to the AI model itself. Section 6 provides a detailed discussion of the proposed taxonomy, including its limitations, and outlines the future directions of the study. Finally, Section 7 presents a summary of the discussed points throughout the paper.

## 2. Related Work

In industrial assembly, human activity recognition requires significant attention due to the critical role humans play in the process [11]. Assembly tasks, characterized by intricate processes and workflows in different fields, are the focus of HAR, which aims to develop models for understanding and classifying these activities, ranging from simple actions to complex, multi-step processes [6]. However, while this categorization assists in assessing complexity, it may overlook factors such as context and granularity. By acknowledging the connection between industrial assembly and HAR, we can explore how insights from activity recognition research can enhance the optimization of manufacturing and assembly operations.

Two main approaches prevail in the literature: binary class and non-binary multiclass approaches, as shown in Table 1, which groups research publications from various domains, such as manufacturing, into recognized activity abstraction categories.

### 2.1. Binary Class Approaches

As mentioned by the authors in [12,13,14,33], compared to **simple activities, complex activities** are composed of actions and are much more complex and semantically consistent with a human’s real life. Ramanujam et al. [48] attempted a division of public HAR datasets that apply deep learning techniques into simple and complex activities, categorizing them into conventional and hybrid models. Nevertheless, the problem remains without the additional distinctions because any task that is more complicated than simple is automatically labeled as complex. Meanwhile, Bouton et al. [47] investigate complex daily activities in sedentary settings such as remote work or study environments, concluding that gaining insights into an individual’s daily activities can help develop applications that improve their well-being and overall health. The work from [37] exhibits higher detection accuracy and less ‘intersubject variability’ as mentioned by the authors, since simple types of activities are performed similarly across users. Additionally, they observed confusion between simple and complex activities such as “cooking and standing” or “sitting on sofa and lying on sofa”. Chen et al. in [44] separate human activities into simple human activities (SHAs) and complex human activities (CHAs), where SHAs may be recognized with an accelerometer, whereas CHAs need multimodal sensor data.

The distinction between activities is crucial in HAR; however, existing taxonomies for human activity abstraction levels are often limited to simple and ambiguous categories that cannot capture the complexity and diversity of human activities across various domains [38,42,43,45,51,52,53,54,55,56,62,63,64,65,66,67]. Zhang et al. in [68] explored complex activities such as eating, which involves a variety of movements, and reported that they increase the challenge and difficulty for HAR methods. For example, an activity such as “cooking” involves actions like “cut”, “take”, or “mix” in different order and frequency. The authors in [16,39] refer to these actions as “micro-activities” or **micro-motions** and characterize the complex activities as “macro activities” or **macro-motions**. In their work, they discuss how micro-motions serve several purposes in understanding macro-motions. This includes (i) confirmation of the execution of all necessary micro-motions; (ii) facilitating evaluation of their sequence correctness; and (iii) recognizing differences among macro-activities or their execution variations.

Different types of activities require different granularity levels, as mentioned by the authors in [34]. In their study, they utilized the terminology “complex activities” of daily living (ADLs), which are built on top of simple activities and convey more specific contextual information, and “simple or coarse-grained activities”, such as walking, sitting, and cycling, which may be directly assessed from an inertial sensor unit.

The terms “coarse-grained and fine-grained activities” and “low-level and high-level activities” are also used to describe different aspects of activity abstraction. The division between **coarse-grained or gross** and **fine-grained activities**,which reflects the level of detail or granularity in activities, appears in industrial manufacturing [15] but also in various other domains [57] including daily activities [40]. Coarse-grained activities are broad categories or high-level actions, while fine-grained activities are more specific and detailed. Fine-grained activities provide a richer understanding of the activity by capturing smaller sub-actions or variations within a broader category. However, this division may not explicitly address complexity or context.

On the other hand, the distinction between **low-level** and **high-level activities** refers to the hierarchical organization of activities [17,18]. Low-level activities are typically microscopic or elementary actions that form the building blocks for higher-level activities. High-level activities encompass a collection of lower-level actions or represent more abstract concepts. This division emphasizes the hierarchical relationship between activities and can be useful for understanding the composition and structure of complex activities. Investigating this topic is interesting since, in most settings, only a few activities are considered (less than 10), and only a few of them discuss hierarchical dependencies between activities on lower and higher levels [69].

Previous research has proposed various levels of activity abstractions, ranging from activities such as walking and sitting to motions such as lifting and grasping. The hierarchical approach to human activity recognition involves recognizing simpler activities initially and using them to recognize higher-level activities. The representation of high-level activities is based on the sub-events or sub-activities that serve as observations derived from the higher-level activity [41]. The use of sub-events not only makes the recognition process computationally tractable and conceptually understandable but also reduces redundancy in the recognition process by reusing recognized sub-events multiple times. An example given by the authors in [41] is that the high-level activity of “fighting” may involve detecting a sequence of sub-events such as punching and kicking interactions.

By distinguishing between activity abstractions, researchers can develop methods or approaches that are tailored to the specific level of abstraction [70,71], thereby improving activity recognition models.

One main finding derived from the authors in [32] is that “activities involving several body parts are more easily recognizable and allow for shorter window sizes”. Yet, they state that the recognition accuracy for such complex activities is still low. This is due to several factors like (i) the inter-class similarity, (ii) the difficulty in defining each activity and its boundaries, and (iii) the lack of open datasets. Furthermore, activity abstractions provide means of interpretability that enable comprehension of the behavior of the assisted user or system. The ability to identify the appropriate level of abstraction is critical for (i) developing effective activity recognition models, (ii) better understanding and gaining insights into human behavior, and (iii) modeling human behavior in a given context.

Several ongoing challenges persist, including issues such as incomplete information within activity datasets, insufficient contextual details accompanying activity data, and the complexities involved in modeling composite activities [6]. Composite activities, as highlighted in the literature by [40,59], pose particular challenges due to their composition of multiple shorter activities and the associated temporal decomposition required for their recognition. Kulsoom et al., in [60], extend the concept of composite activities and present additional categorizations based on operation types, namely concurrent, sequential, and interleaved activities. Moreover, despite the growing interest in interaction recognition, the recognition of activities involving groups of people [30] has received relatively less focus, as identified by Morshed et al. [61].

In their work, the authors in [72] state that “the existing taxonomies in the field of activity recognition, while valuable, exhibit limitations in their categorization by not encompassing sufficient distinct activity categories, thereby indicating the need for further refinement and improvement”. The presented studies provide evidence of the prevalence of binary distinctions and the limitations in existing methodologies. Thus, describing activities as simple or complex, high or low level, and fine or coarse-grained hinders a detailed enough understanding of the underlying components and structures of human activities, the different sub-components of activity, and how they relate to each other. Therefore, there is a research gap in exploring beyond the simple–complex dichotomy, which can be addressed by incorporating additional levels of activity abstraction.

### 2.2. Multiclass (Non-Binary) Approaches

The hierarchical framework provided by Kuutti et al. [73] offers insights to address the limitations of binary classifications in human activity recognition. In this, the authors explain how activities consist of chains of actions, with each action comprising individual operations, where individual actions become comprehensible only when viewed within the larger context of the activity. Recent findings by Miranda et al. [46] suggest that a promising strategy for dealing with complex HAR is to model activities as a series of dependent atomic actions. In this context, Saguna et al. [50] focus on the semantics of the application and explain it as a fundamental activity unit that cannot be further decomposed.

Following the previous explanation and using comparable methods in the existing literature, we refer to the framework of **Atomic Actions, Primitive Tasks, Tasks** that comprises three hierarchical levels: task, primitive task, and atomic action [19,20,21,22,23,74]. An assembly task is decomposable into multiple continuous primitive tasks, which can be executed sequentially or in parallel. Similarly, each primitive task can be further broken down into continuous atomic actions, which are also capable of sequential or parallel execution. While the proposed framework addresses the binarization encountered in the literature regarding activities, our approach proposes different classifications for levels or actions involved in certain activity stages, respectively. The proposed taxonomy provides a more refined classification for some actions than existing frameworks to cover the complexities of human activity in industrial contexts. We believe that certain actions, as defined in their context, can be further decomposed or integrated into a broader hierarchy of activities.

The challenge in activity recognition lies in the generalization of models across diverse contexts [65] through recognizing patterns in sensory input. Conventional approaches often oversimplify activities as either simple or complex without accounting for contextual variations. Furthermore, human activity recognition tends to be associated with locomotion activities, and often instead of employing categorizations based on factors like complexity or granularity, many studies opt for a broad approach to activities without specific classification [25,27,28,29,31,58]. An additional aspect was addressed by the authors in [36] where they provide insight into the characteristics of complex activities, highlighting that complex activities exhibit longer duration, comprise a combination of simple activities, and encompass multiple behaviors. These complex activities often possess high-level semantics, such as daily activities like cooking, cleaning, or industrial assembly tasks.

Examining binary and multiclass approaches revealed that certain categories of activities often receive less attention, as they are often processed using the same models designed for other categories. However, these activities are important in the recognition and classification process and require customized methods due to their distinct attributes and complexities. Hence, it is critical to distinguish the nuanced differences between activities across various contexts and categorize them appropriately to leverage context-specific characteristics for understanding user behavior to improve activity recognition systems in real-world settings.

## 3. Proposed Taxonomy Model

We propose a taxonomy using a hierarchical activity abstraction structure that combines the simplicity of the simple–complex division with the clarity of the non-binary approaches to study activities at different levels of granularity and abstraction along with their respective sub-divisions. Additionally, our work extends beyond taxonomy development to offer practical applicability and relevance in real-world industrial scenarios.

### 3.1. Industrial Assembly Process

In industrial manufacturing, assembly processes are essential for joining multiple components to produce functional products [11]. Whether it is assembling automobiles, electronic devices, or machinery, the efficiency and effectiveness of the assembly process impact the success of the manufacturing operation. Workflow optimization depends on an understanding of the hierarchical structure and relationships between different phases of assembly. Every stage of the assembly process on a manufacturing floor, typically involving a frame (e.g., chassis) where modules are systematically assembled to construct complex products, requires coordination, precision, and adherence to defined work instructions. In [75], the authors describe the hierarchical nature of complex product assembly data, highlighting three granularity levels of product, assembly, and part. They state that the refined management of assembly processes and hierarchical organization of assembly data can be achieved by decomposing complex assembly activities into more detailed activities.

Building upon this framework, our analysis of real-world assembly processes revealed the hierarchical structure observed in complex assembly scenarios [11,75,76,77]. Moreover, our analysis aligns with findings from the literature regarding the roles of modular assembly and units or sub-assemblies in the assembly process. By breaking down complex assembly tasks into modular units, manufacturers can enhance efficiency and flexibility in their production workflows, separate design tasks into distinct units, and simplify the design process while prioritizing product customization [78,79] and responsiveness [80]. The authors in [81] highlighted the importance of the number of modules, joining sequences between modules, and tolerance management issues in car body design. In assembly processes, modules are self-contained subsystems designed to be constructed, examined, manufactured, and developed independently from the overall system for independent integration. This ensures interchangeability, standardization, and re-usability, with clear interfaces and relative independence from other modules [82].

**Figure 1:**
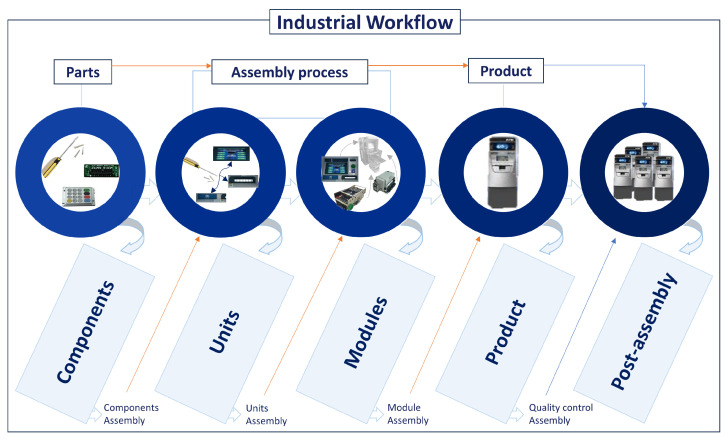
This figure presents a hierarchy of an exemplary assembly process: components, units, modules, products, and post-assembly. Additionally, it demonstrates how these stages are interconnected and how activities and tasks flow inside a real assembly scenario from components to the final product. Each stage builds upon the previous one, with components being assembled into units, units into modules, modules into the final product, and finally, the product being integrated into the production line.

In addition, our work recommends adding the post-assembly processes [83] as a level to the assembly process hierarchy where the concept of the final product is contextualized within the scope of the specific manufacturing line. This indicates that what constitutes the final product may vary depending on the manufacturing line. For instance, what serves as the final product on one manufacturing line might be considered a sub-assembly or component in another line. With this feature, industrial processes are observed, covering every stage of the manufacturing process, from components to finished products, by performing the required manual activities for each stage. Therefore, the assembly chain example presented in Figure 1, for understanding various industrial workflows, does not extend to subsequent stages of product development or distribution but focuses on the assembly process of a product, which is completed on the production line.

### 3.2. Abstraction Levels of the Taxonomy Framework

**Figure 2:**
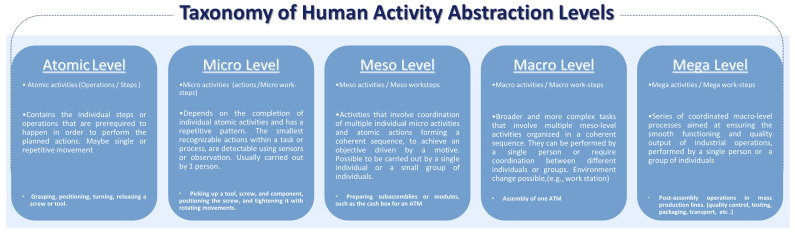
The figure presents a visualization for the proposed taxonomy. At the atomic level, individual assembly activities are considered as singular tasks involving basic operations or manipulations on discrete components or tools. The micro-level aggregates multiple atomic operations into coherent sequences, representing actions within the assembly process. Larger assembly tasks are formed at the meso-level by combining multiple micro-level activities, often involving the assembly of sub-components or partial assemblies. The macro-level encompasses entire assembly processes, including stages such as the assembly of major components or modules. Finally, the mega-level represents the overall assembly process, incorporating post-assembly activities such as quality control checks, packaging, or final inspection.

The proposed taxonomy presented in Figure 2 introduces five stages—atomic, micro, meso, macro, and mega—providing a hierarchical framework for analyzing and categorizing activities within the assembly process of discrete manufacturing products to understand the progression of assembly tasks. In our framework, we employ the term “activities” for the different levels to ensure consistency, adopting a unified terminology throughout our analysis. With the systematic use of the terms atomic, micro, meso, macro, and mega, we denote the distinctions of activities at various stages of the assembly workflow.

**Atomic**: This level includes the smallest self-contained operations or steps that can be performed by a human in a manual product assembly scenario involving the basic components or tools for discrete or singular manipulations within a broader action or activity. Examples of such atomic operations or sub-tasks that contribute to completing a particular action could be grasping the screw or tool, positioning the screw or tool, turning the screw or tool, and releasing the screw or tool.

**Micro**: This level includes the smallest recognizable actions within a task or process that serve a specific goal, detectable using sensors or observation, requiring tools to execute certain tasks like joining or attaching components. They are composed of a sequence of atomic steps, generating singular activities, including operations that can be executed by a single individual without extensive planning or coordination. They are characterized by their relatively short duration and repetitive pattern, and they can be performed independently or as part of a larger activity as an individual work step. At the micro-level of assembly, individual tasks are executed by integrating multiple atomic operations, each involving distinct components, tools, or parts. The sensor data required to detect screwing as an activity can be limited to the movement and orientation of the tool, as this information alone contains indicative signals of the screwing action. An example of a micro-activity is the entire process of picking up a tool, a screw, and a component, positioning the screw in the part, and using the tool with rotating movements to tighten a screw.

**Meso**: This level includes a collection of coordinated actions and operations from the micro-level and/or atomic level, forming a coherent sequence to achieve an objective driven by a particular motive. This level bridges the gap between fundamental atomic and micro-level processes toward increasingly complex processes, representing a crucial stage in assembly workflows to accomplish intermediate goals typically undertaken by a single individual or a small group of individuals. At the meso-level, sub-assemblies, and modules of the product are prepared through the aggregating micro-level activities, which are each designed with specific functions and aims. They are self-contained units that meet intermediate ends, which are essential steps toward the final assembly. An example of a meso-level task would be to prepare the sub-assemblies of a module, such as the cash box for an ATM.

**Macro**: At the macro-level, tasks evolve into broader and more complex tasks, encompassing multiple steps and components from the previous meso-level. This process involves coordinating multiple meso-level activities, which include the assembly of sub-assemblies and modules prepared in earlier stages. Macro-activities can involve sequences of actions, including those demanding higher-level cognitive functions like decision making and planning. These tasks might require coordination between individuals, groups, or machines (robots) where the outcome of this coordination results in the accomplishment of a complex goal, such as the full assembly of a product (e.g., the ATM) depicted in Figure 1.

**Mega**: This level comprises a series of coordinated macro-level processes to ensure the smooth functioning and quality output of industrial operations. In a broader sense, mega-level activities may involve various tasks conducted by humans beyond the assembly line operation yet included in the industrial workflow. These could include packaging products for safe transport and inspection, testing functionality, or conducting quality-control checks. These activities contribute to the overall goal of achieving efficient and effective production processes on a large scale. Examples include the post-assembly operations that exist in mass production lines of individual products, such as cars or other complex items assembled from multiple modules or sub-assemblies.

Following the presented hierarchical structure of complex processes in industrial manufacturing, focusing on the assembly of products, we connect these concepts with the industrial example illustrated in Figure 1. This example provides an overview or a reference to our approach rather than a comprehensive representation of all applications included in an industrial setting. In the assembly of automated teller machines (ATMs), the hierarchical structure of assembly processes is evident across separate abstraction levels. At the component level, where atomic activities are involved, screws, tools, and individual parts like display panels form the foundational building blocks of the ATM. These components are then manipulated and used to create the required sequential work steps that contribute to developing units at the micro-level. They often involve repetitive tasks such as tightening motions with rotating movements using tools. While the micro-level focuses on one specific single action every time, the meso-level relies on the coordination of multiple actions to complete larger modules like cash-handling systems or user interface systems that are assembled independently during that level. All modules, units, and the remaining components are assembled into a complete ATM system at the product or macro-level. Subsequently, in the post-assembly stage, which represents the mega-level, the final product is integrated into the manufacturing line, undergoing additional processes such as quality control, software installation, functionality testing, and packaging.

In addition to the hierarchical structure demonstrated within the ATM assembly, our activity recognition taxonomy extends beyond this specific application to cover various industrial contexts. To validate its versatility, we have developed a hierarchical formulation that represents assembly processes using equations. This conceptual representation, through equations, serves as an initial tool for our activity recognition taxonomy, providing insights into the intricacies of assembly tasks to understand the relationships between activities within industrial assembly workflows. In this model for assembly processes, we utilize a set of symbols to quantify various attributes at different hierarchical levels. The specific step of the assembly activity, performed at a given level, is indicated by the symbol $A_{\mathrm{level}}$, and the time spent on each step of the activity is indicated by the symbol $t_{\mathrm{level}}$. Moreover, $a_{\mathrm{level}}$ represents the kinematic characteristics associated with the assembly step or operations at that level, like acceleration or angular velocity. The equation $P_{\mathrm{level}}$ expresses the overall occurred activities at a specific level within the taxonomy framework. Throughout the model, summations aggregate the contributions of individual activities, providing an analysis of assembly workflows.

**Hierarchical formulation of our taxonomy in an assembly process**

*Attributes:*

- $P_{\mathrm{level}}$: Activity on level of taxonomy.
- $A_{\mathrm{level}}$: Step of assembly activity.
- $t_{\mathrm{level}}$: Time spent on step of assembly activity.
- $a_{\mathrm{level}}$: Kinematics properties on step of assembly activity

$$P_{{\mathrm{atomic}}_{i}}=f\left(A_{i},t_{i},a_{i}\right)$$

$$P_{{\mathrm{micro}}_{j}}={\left\{P_{{\mathrm{atomic}}_{j}}\right\}}_{j=1}^{n}$$

$$P_{{\mathrm{meso}}_{k}}=\left\{P_{{\mathrm{micro}}_{k}}\right\}\cup {\left\{P_{{\mathrm{atomic}}_{k}}\right\}}_{k=1}^{m}$$

$$P_{{\mathrm{macro}}_{l}}=\left\{P_{{\mathrm{meso}}_{l}}\right\}\cup \left\{P_{{\mathrm{micro}}_{l}}\right\}\cup {\left\{P_{{\mathrm{atomic}}_{l}}\right\}}_{l=1}^{p}$$

$$P_{{\mathrm{mega}}_{q}}=\left\{P_{{\mathrm{macro}}_{q}}\right\}\cup \left\{P_{{\mathrm{micro}}_{q}}\right\}\cup \left\{P_{{\mathrm{PA}}_{q}}\right\}\cup {\left\{P_{{\mathrm{atomic}}_{q}}\right\}}_{q=1}^{r}$$

## 4. Comparative Analysis with SOTA

**Figure 3:**
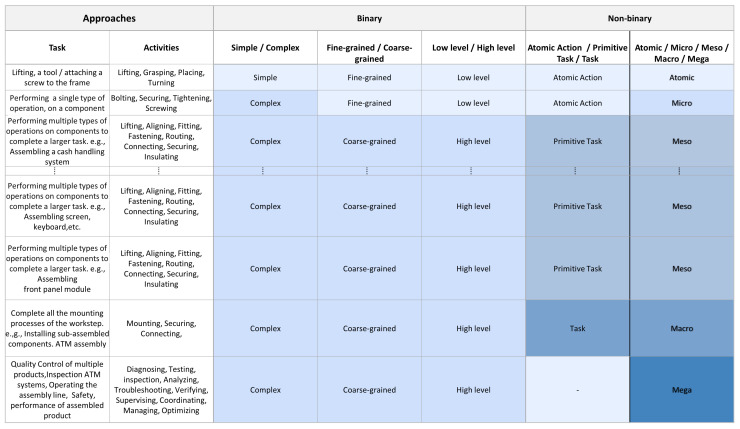
The table illustrates a simplified ATM assembly process, derived from a real industrial use case [76], showcasing activities across different assembly levels: atomic, micro, meso, macro, and mega. It serves as a comparative analysis with existing approaches for activity categorization, highlighting how each level contributes to the overall process. Specific activities are provided for clarity, offering insights into the hierarchical organization of assembly tasks. The color coding highlights differences in categorization when distinguishing tasks across levels.

In this section, we compare our introduced taxonomy to the current state-of-the-art (SOTA) approaches that exist in the literature, and in the following section, we present the application of our taxonomy to real-world industrial scenarios. After reviewing relevant publications outlined in Section 2, we generated a categorization methodology for an assembly scenario to visualize the differences between binary and non-binary approaches and the proposed one. In Figure 3, we present a simplified process assembly of an ATM illustrating some key steps that capture a subset of the entire assembly, as the complete one typically involves numerous additional steps and complexities. This example is derived from original scenarios and real-world use cases documented in our prior work [66,70,71,76,84], discussing ATM and digger assemblies in more detail.

In simple/complex, fine-grained/coarse-grained, low-level/high-level classifications, we observe a two-stage categorization method between detailed and broader classifications as outlined in our related work. These approaches emphasize the distinction between the initial assembly tasks as sub-actions and their combination as broader categories. For instance, in the simple/complex approach, a screwing process already belongs to the complex category, since it is considered much more complicated compared to lifting a hand or grabbing an object. Additionally, in the fine-grained/coarse-grained, low-level/high-level categories, lower-level classifications consist of activities similar to screwing, as the primary stage, and continue afterward to more demanding activities.

At the atomic level, activities involve manipulating individual tools and components through discrete or singular manipulations. These actions serve as the building blocks for more complex tasks. For instance, lifting a tool or grasping a single screw constitutes an atomic action. Moving to the micro-level, tasks involve the execution of specific actions that combine interactions between at least two elements from the atomic level to form a single type of action. For example, screw tightening involves grasping, lifting, and turning a screw with a tool. The micro-level is characterized by repetitive actions, such as repeatedly rotating a screw to fix it tight. At this level, a difference can be noticed between the proposed methodology and the non-binary approaches reported in the literature. In our methodology, the screwing process occupies a different level compared to lifting or grasping because it is considered a combination of those actions.

**Figure 4:**
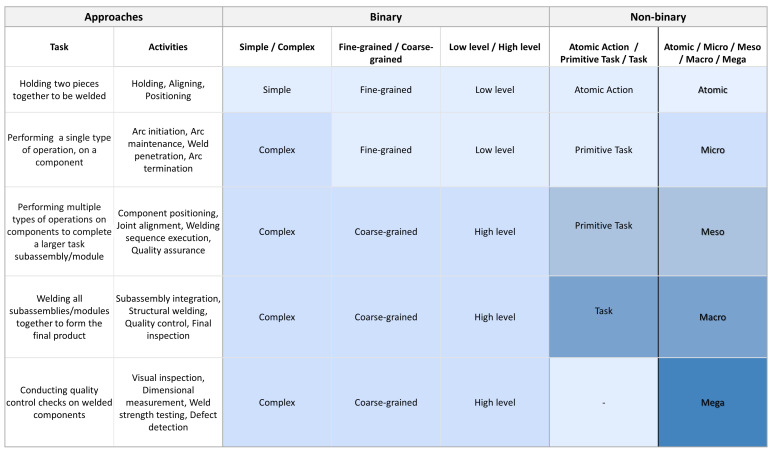
The table illustrates welding processes in car assembly, presenting the hierarchical framework of tasks, and showcasing activities across different assembly levels: atomic, micro, meso, macro, and mega. It serves as a comparative analysis with existing approaches for activity categorization, highlighting how each level contributes to the overall process and showing how individual actions aggregate into more complex tasks across the assembly line. Specific activities are provided for clarity, offering insights into the hierarchical organization of assembly tasks. The color coding highlights differences in categorization when distinguishing tasks across levels.

Transitioning to the meso-level, activities become more comprehensive, involving the combination of micro-level tasks to accomplish intermediate goals. For instance, assembling an ATM’s front panel system requires the integration of micro-level activities. At the macro-level, larger components and subsystems, previously prepared at the meso-level, are brought together to construct the final product. This step involves coordinating multiple meso-level activities, each contributing essential components or modules designed with specific functions and aims. For example, assembling the ATM at the macro-level entails integrating pre-assembled sub-parts, such as the cash-handling system and front panel to the chassis into a cohesive structure. In our methodology, we view the assembly process as an integral part of a larger manufacturing ecosystem rather than an isolated event. In this regard, at the mega-level, the focus extends beyond assembly to encompass broader activities such as quality control, inspection, and overall process optimization, as opposed to existing approaches that often overlook such human tasks. In the second example of an industrial assembly process, the focus shifts from screwing processes to welding processes within the context of car assembly. This hierarchical framework presented in Figure 4 remains the same, including the five levels: atomic, micro, meso, macro, and mega, illustrating the progression of tasks.

## 5. Application of the Taxonomy for Guiding AI System Design

**Figure 5:**
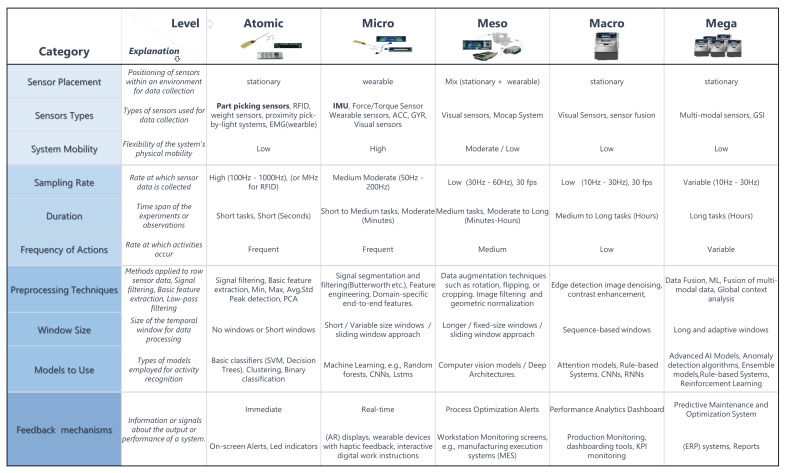
This figure illustrates key characteristics across atomic, micro, meso, macro, and mega-levels of assembly activity recognition systems. Each group of related elements is color-coded, and each line represents a different category, ensuring distinctions between aspects. The figure highlights variations that are important in the overall design of an AI system, such as sensor placement, types of sensors used, system mobility, sampling rate, duration of experiments, frequency of actions, preprocessing techniques, models employed for activity recognition, window size for data processing, and feedback mechanisms. Associated recommendations are provided for each category and level to serve as a starting point for the development of AI models under the “Models to Use” category, which is related to industrial assembly.

In this section, we proceed to the practical application of our taxonomy by presenting its deployment in real-world industrial scenarios. In our analysis, we identify and propose key categories to guide or support practitioners in the research and implementation of AI applications and systems in industrial assembly processes, as previously presented in Figure 5. We provide recommendations for designing AI systems customized to meet the specific requirements of individual use cases and address the design of an AI system as a collective contribution of multiple elements beyond just the AI model itself. These elements encompass but are not limited to (i) sensor placement, sensor types, and sensor mobility [7,60,83,84,85,86,87,88,89,90,91,92,93,94,95,96,97,98,99,100,101,102], (ii) sampling rate, duration, frequency of actions [66,76,103,104,105,106,107,108,109], (iii) preprocessing techniques, window size, models to use [8,24,26,35,94,97,103,109,110,111,112,113,114,115,116,117,118], and (iv) interaction and feedback mechanisms [93,119,120,121,122,123].

At the atomic level, where activities involve individualized tool interactions and component manipulations, sensors are typically mounted on the parts, components, or body, ensuring the capture of object movements and interactions. These identification sensors (ID) encompass a diverse range, including RFID, weight sensors, proximity-aware, pick-by-light systems, or EMG, allowing for multifaceted data collection. Preprocessing techniques applied to raw sensor data involve signal filtering, feature extraction, peak detection, and PCA approaches, which, in the scope of our work, are selected for refining the data for subsequent analysis. Machine learning models, such as basic signal processing and classifiers like SVMs and decision trees, are employed to recognize basic manipulation tasks characteristic of this level, sometimes without the need for temporal windows.

Transitioning to the micro-level, where activities entail more specific tasks involving interactions with parts from the previous atomic-level, a similar sensor setup is maintained with an increased focus on hand manipulations and sequences of atomic-level tasks. Preprocessing techniques, if applied, become more refined, incorporating signal segmentation and feature engineering within time-series data to capture sequential actions. Machine learning models evolve to include CNNs and RNNs capable of analyzing more complex interactions, while short or adaptive windows facilitate the analysis of sequential actions and interactions. Despite these advancements, the focus remains usually on single users engaging in short-duration tasks with high-frequency actions.

As we progress to the meso-level, activities involve performing specific tasks using larger components, which are typically occurring at the main assembly station or sub-assembly stations. Sensor placement may extend to encompassing the assembly workstation, capturing interactions between larger components or modules. In order to facilitate comprehensive data capture, vision sensors, depth cameras, and Mocap systems are introduced. In addition, preprocessing techniques become more sophisticated and memory extensive, incorporating feature extraction, windowing, augmentation techniques, image filtering, and object recognition. Computer vision and deep learning models, such as CNNs and LSTMs, are employed to recognize complex assembly patterns with longer or adaptive windows enabling the analysis of entire assembly sequences and sub-tasks.

At the macro-level, where activities encompass complete assembly processes and integration tasks, the sensor setup may cover the entire assembly workstation or multiple adjacent workstations, combining sensor systems [102] to capture interactions across multiple sub-assemblies or components. This level requires advanced AI models, ensemble models, and rule-based systems to analyze complex assembly workflows effectively. Long and adaptive windows, depending on the type of employed data, facilitate the analysis of entire assembly processes and integration tasks, while the subject number may extend to multiple users or groups engaged in longer assembly processes and system integration.

Finally, at the mega-level, activities involve comprehensive manufacturing processes and the optimization of production workflows across the entire factory floor or assembly facility. Sensor placement becomes more varied, capturing interactions across the entire facility, while preprocessing techniques involve global context analysis, advanced machine learning, and data fusion techniques. Advanced AI models, deep learning architectures, and custom ensemble models are employed to analyze complex manufacturing processes and optimize production workflows. Variable window sizes and sampling rates are tailored to capture diverse manufacturing activities and system performance metrics across the facility with infrastructure supporting real-time data processing and the optimization of manufacturing operations.

## 6. Discussion

The employment of our multiple-level activity abstraction scheme offers several compelling benefits. Firstly, it facilitates a better understanding of complex systems by breaking them into smaller-level activities. This breakdown allows researchers and industry professionals to gain insights into the individual components that constitute the larger system, leading to a more detailed understanding of workflows, training programs, and automation systems. Consequently, it becomes easier to identify areas for improvement, troubleshoot problems, and optimize overall system performance. Apart from that, recognizing such complex activities is also important for daily living activities because it can enable tracking digital well-being, providing context-aware user experiences and notifications, and allowing better content recommendations [34].

In our understanding of assembly processes, certain activities may appear similar to atomic tasks but differ in nature and purpose. For instance, while activities like walking between workstations or reading assembly instructions are essential, they are either locomotion activities or cognitive tasks rather than physical manipulations of components. Although locomotion activities are inherently part of the overall activity framework in assembly tasks, individuals typically maintain a static posture while executing them, focusing on manipulation and precise task execution over physical movement. On the other hand, operating machinery or inspecting finished products involves complex activities beyond the scope of atomic actions. These tasks encompass a range of actions, such as controlling machinery or evaluating product quality, that require cognitive judgment and coordination. While they contribute to the overall process, they are distinct from the discrete singular actions associated with atomic tasks that would be under the latter stage in our hierarchy.

Within the domain of human activity recognition in industrial assembly tasks, the ability to recognize micro-activities, such as screwing, without the requirement to detect separately every underlying sub-action emerges as an interesting aspect. This is due to the goal-oriented nature and foundational role that micro-activities play in the hierarchical structure of assembly processes. Even though a micro-activity can be divided into smaller steps, recognizing the indicative pattern of the micro-activity reduces the need to understand separately the independent atomic actions except when tasks specifically require identifying manipulations at the atomic-level. The rationale for this is that detecting all the sub-actions would require more sensors and computing capacity, which might not essentially provide more meaningful information about the task at hand. Instead, the focus is on detecting the overall activity and its characteristics (such as duration, frequency, etc.), which can be captured using a smaller number of sensors and analyzed more efficiently while indicative of the underlying atomic-level components.

Regarding the duration of activities, each level exhibits a distinct timescale in a particular scenario. Micro-activities, which are characterized by repetitive actions, typically have shorter duration. As activities progress to higher levels, incorporating multiple previous activity levels, the duration increases proportionally, reflecting the cumulative complexity and scale of tasks involved. For instance, a meso-level activity comprising 10 micro-activities has a longer duration, which is calculated by aggregating the duration of each micro-level and atomic-level activity. This temporal progression also highlights the hierarchical nature of assembly tasks and underscores the incremental development of products across different levels. Nevertheless, there may be some slight overlap in the activity duration due to the variety of activities in the industry. Therefore, while the timescale can indicate each level, scenario analysis is required for interpretation.

Additionally, atomic, micro, meso, macro, and mega-level activity abstractions contribute to a common understanding that bridges the gap among people of diverse roles and expertise in the assembly process, facilitating effective communication and knowledge transfer to achieve greater productivity, execution accuracy, and scalability. Each successive abstraction level builds upon actions that exhibit variations, such as the number of individuals involved, the execution station utilized, the overarching goals they serve, and the complexity of the tasks they encompass. For instance, at the atomic or micro-levels, activities may involve individual actions such as grasping a component or tightening a screw. These actions are relatively simple and executed by individual workers at specific workstations. As we move to higher levels such as the meso or macro and mega-levels, micro-activities become more complex, involving coordination among multiple workers or machines across different stations to achieve larger production goals. Furthermore, the end goals of micro-activities differ across levels. At the micro-level, the focus may be on completing discrete assembly tasks, whereas at the macro and mega-levels, micro-activities contribute to broader objectives such as optimizing production efficiency or meeting customer demand.

The hierarchy of assembly stages can also benefit both the arrangement of sensors and data analysis. By grouping the assembly process into distinct stages, we can deploy wearable sensors, such as IMUs or visual sensors, to gather relevant information. These stages can provide real-time data awareness of worker movements and interactions with components or data collection tools at each level. In addition, researchers can identify and consider more factors related to specific cases. For instance, experiments at the atomic level may occur in controlled lab environments, ensuring precise data collection. However, as assembly complexity evolves at the macro and mega-levels, experiments transition to real-world factory settings, introducing factors that may include limited lighting, occlusion effects, noise, and other environmental variables along with the system’s obtrusiveness. The complexity of tasks also impacts the frequency of actions, with activities occurring more frequently at lower assembly levels, such as the atomic and micro-levels, compared to the macro and mega-levels, where actions occur less frequently. Additionally, privacy concerns rise with increasing complexity reflecting the diverse and extensive nature of data collection across the factory floor while underscoring ethical and practical considerations in system development. Addressing potential data privacy implications led us to prioritize the selection of less invasive and privacy-friendly sensors for the applicable levels, considering the individual’s privacy rights.

These differences underline the need for tailored development measures and regulations compliance, as assembly activities progress from lower to higher levels of complexity, which plays a significant role in user acceptance, system deployment, and overall effectiveness in real-world assembly environments. Moreover, considerations surrounding data processing and power consumption grow across assembly levels. While at the atomic and micro-levels, data processing and sensor cost are low, progressing to the macro and mega-levels, there is an increasing requirement for computational and finance resources, especially if the generated data will be stored.

Overall, it is reasonable that while we try to create a versatile and robust framework, there may be limitations to covering each case in all different domains of HAR. Consequently, we present our method for industrial assembly processes, but it may need adjustments to describe activities in other domains. Additionally, some specific use cases may fall within the boundaries of the described levels. In such cases, we recommend drawing recommendations from both levels and addressing with a hybrid approach the complexities of the case. Although extensive, we acknowledge that these recommendations cannot serve as the sole method or rigid rules due to the individual requirements, challenges, and outcomes inherent to each unique case. However, they provide a valuable starting point and a preliminary framework for navigating the complexities of designing and implementing AI technologies in industrial settings.

Our future work focuses on the emerging trends and paradigms shaping the landscape of human interaction with advanced technologies beyond the manufacturing sector. Addressing the complexity of activities within HAR while extending hierarchical analysis to diverse domains will be a priority to create a more general framework that can include multiple activities. Moreover, these categorizations of activities may support the research and development of activity datasets and models that specifically address the complexities of each level. Additionally, it is mandatory to recognize the importance of safety within industrial environments [124,125], while improving the system’s interoperability and the user’s experience, making assembly activity recognition systems more human-responsive. This means creating systems that understand and respond to human needs and behaviors better. One example would be to develop technologies that detect when a worker needs help, is feeling stressed, or is feeling fatigued, and assist or adjust the workload accordingly. It is therefore important to investigate how individuals interact with assistive technology (e.g., smart products, collaborative robots) for assembly tasks. Investigation of this socio-technical aspect is already present, e.g., in [126,127]. By exploring these insights in future research and development, we can advance the capabilities of assembly activity recognition systems, improve communication between humans and machines, promote teamwork, and facilitate more efficient and effective operations in industrial environments, while manufacturing processes can become more efficient.

## 7. Conclusions

Our work aims to enhance the understanding and support of human activities within industrial contexts. To achieve this, we developed a comprehensive taxonomy, that spans from the atomic to the mega-levels, for categorizing human activities in assembly processes for discrete product manufacturing. This taxonomy offers a hierarchical structure that facilitates better decision making, process optimization, and system design that addresses the binarization of <<simple>> and <<complex>> activities. We demonstrate the differences in the approaches with a comparison between the proposed taxonomy and the existing categorization schemes through an example of a real assembly scenario. By breaking down tasks into distinct levels, we enable a more granular analysis, identifying areas for improvement, troubleshooting problems, and optimizing the overall system performance.

Building upon this taxonomy, we provide specific recommendations for designing AI systems tailored for activity recognition in industrial assembly tasks. Although each use case may be unique with specific requirements and goals, the fundamental human activities and physical manipulations of the tools and components across assemblies typically remain similar. These recommendations examine, among others, sensor placement, preprocessing techniques, and model selection across different levels of activity abstraction.

Hence, it is important to align the crucial role of sensor systems attributes with specific application requirements to optimize the design of an AI system while ensuring both performance and functionality are effectively maintained. By leveraging the taxonomy, we aim to support the development of reliable, robust, and suitable for real-world deployment AI systems besides facilitating effective communication between experts and non-experts.

Overall, our research contributes to advancing the capabilities of AI-driven systems in industrial settings, fostering more efficient, safe, and human-responsive manufacturing processes. By providing a structured framework for analyzing and understanding human activities and offering recommendations for AI system design, we seek to empower researchers and professionals in various industrial fields to develop more effective methods and tools for studying, modeling, and supporting human activities.

## Figures and Tables

**Table 1:** This table presents an overview of the existing literature in the domain of human activity recognition, presenting a quantitative distribution of papers across different abstraction levels and domains. The dominance of publications at the simple/complex level across various domains suggests a significant focus on understanding activities in this distinction. On the other hand, the comparatively small number of publications in some areas, such as group activities, suggests possible topics for more investigation and study. Furthermore, the existence of publications at various levels of abstraction highlights the complexity of human activity recognition research and emphasizes the necessity for sophisticated methods of activity analysis and classification.

Abstraction Levels							
**Approaches:**	**Binary**				**Non-Binary**		
**Domain**	**Atomic-Simple, Complex-Composite**	**Composite-Gross, Fine-Grained**	**Micro, Macro**	**Low, High-Level**	**Gestures, Actions, Interactions, Group Activities**	**Atomic action, Primitive Task, Task**	**Various, Other Terminology**
Manufacturing, Robotics, Construction	[12,13,14]	[15]	[16]	[17,18]		[19,20,21,22,23,24]	[25,26]
Healthcare							[27,28,29]
Sports					[30]		[31,32]
ADLs	[9,10,33,34,35,36,37,38]			[33]			
IADL	[37,39]	[40]	[39]				
Group					[41]		
Other Domain, Unspecific HAR	[6,8,36,38,42,43,44,45,46,47,48,49,50,51,52,53,54,55,56]	[57]					[58,59,60,61]

## Data Availability

Skoda public dataset for Human activity recognition.
